# Exploring the Relationship between Acute Coronary Syndrome, Lower Respiratory Tract Infections, and Atmospheric Pollution

**DOI:** 10.3390/jcm13175037

**Published:** 2024-08-25

**Authors:** Paweł Muszyński, Elżbieta Pawluczuk, Tomasz Januszko, Joanna Kruszyńska, Małgorzata Duzinkiewicz, Anna Kurasz, Tomasz A. Bonda, Anna Tomaszuk-Kazberuk, Sławomir Dobrzycki, Marcin Kożuch

**Affiliations:** 1 Department of Invasive Cardiology, Medical University of Bialystok, M. Skłodowskiej-Curie 24A, 15-276 Bialystok, Polandmarcin.kozuch@umb.edu.pl (M.K.); 2 Department of General and Experimental Pathology, Medical University of Bialystok, Mickiewicza 2C, 15-230 Bialystok, Poland; 3 Department of Cardiology, Lipidology and Internal Diseases, Medical University of Bialystok, Żurawia 14, 15-569 Bialystok, Poland; kardiologia.lipidologia@umb.edu.pl

**Keywords:** air pollution, coronary thrombosis, acute coronary syndrome, respiratory infections

## Abstract

**Background**: Respiratory infections were found to be connected with the incidence of acute coronary syndrome (ACS). The proposed pathway of this connection includes inflammation, oxidative stress, pro-coagulation, and atherosclerotic plaque destabilization. This can cause rapture and thrombus formation, leading to ACS. Our study aimed to assess the risk factors for coronary artery thrombosis as a manifestation of ACS and for lower respiratory tract infections (LRTIs) in patients with ACS. **Methods**: The study included 876 patients with ACS from January 2014 to December 2018. Both the clinical data and air pollution data were analyzed. Statistical tests used for analysis included Student’s *t*-test, the Mann–Whitney U-test, the Chi-squared test, and the odds ratio Altman calculation. **Results**: LRTIs were found in 9.13% patients with ACS. The patients with LRTI had a higher risk of coronary artery thrombosis (OR: 2.4903; CI: 1.3483 to 4.5996). Moreover, they had increased values of inflammatory markers, were older, had a lower BMI, and a higher rate of atrial fibrillation. The average atmospheric aerosols with a maximum diameter of 2.5 μm (PM_2.5_ concentration) from three consecutive days before hospitalization for ACS were higher in patients with LRTI. **Conclusions**: The occurrence of coronary artery thrombosis was higher among the patients with LRTI during ACS. PM_2.5_ exposition was higher in the three consecutive days before hospitalization in patients with LRTI during ACS.

## 1. Introduction

Acute coronary syndromes (ACSs) are a group of conditions that include ST-elevation myocardial infarction (STEMI), non-ST elevation myocardial infarction (NSTEMI), and unstable angina (UA). ACSs arise as a manifestation of coronary heart disease (CHD) and typically result from the destabilization of the atherosclerotic plaque in the coronary artery, leading to its dynamic narrowing or occlusion [1]. Coronary artery thrombosis, leading to ACS, can be caused by plaque rapture, erosion, or calcified nodules [2]. The main plaque-destabilizing factors are endothelial injury and local inflammation. This can be driven by common CHD risk factors like smoking, hypertension, diabetes mellitus, or hyperlipidemia. This can also be supported by remote or systemic inflammation that is entangled in non-classical CHD risk factors like exposure to air pollution or chronic obstructive pulmonary disease (COPD) [1,3]. Despite the decrease in the prevalence of ACS, especially in high-income countries, ACS remains the main health issue leading to premature death and disability [4]. There is a need for further research to improve prevention tools.

Ambient air pollution is one of the major environmental health problems, leading to 4.2 million premature deaths worldwide in 2022, according to the World Health Organization (WHO) [5]. Air pollutants include particulate matter (PM)—inhalable substances composed of different compounds: sulphate, nitrates, ammonia, sodium chloride, black carbon, mineral dust or water, nitrogen dioxide (NO_2_), ozone, carbon monoxide (CO), sulfur dioxide (SO_2_), lead, polycyclic aromatic hydrocarbons, formaldehyde, and radon. Smog in Poland is formed under high atmospheric pressure and negative air temperature, which differs from Los Angeles and London smog types. Bialystok, where the air pollution data were obtained, is located in Eastern Poland and has a continental climate with cold, long-lasting winters and predominantly warm summers [6].

Evidence from extensive epidemiological and experimental studies supports the relationship between PM exposure and cardiovascular disease (CVD) events. Reductions in PM_2.5_ levels are associated with a decreased severity of inflammation, thrombosis, oxidative stress, and fatalities from ischemic heart disease. However, the exact pathomechanisms remain unclear. In parallel, PM exposure increases the risk of respiratory diseases like COPD, or lower respiratory infections [7]. PM affects the integrity of the respiratory epithelial barrier, impairs the hosts’ defense and mucosal clearance mechanisms, and promotes infections, leading to an exaggerated inflammatory response [8,9]. 

Lower respiratory tract infections (LRTIs) are commonly caused by bacteria such as Streptococcus pneumoniae, Haemophilus influenzae type b, and viruses like respiratory syncytial virus or influenza, and in recent the period, by severe acute respiratory syndrome coronavirus 2 (SARS-CoV-2). Acute infections share some inflammatory pathways with atherosclerosis. The respiratory infections activate innate immune responses that promote endothelial dysfunction and atherosclerotic plaque instability, boost blood hypercoagulability and support coronary artery thrombosis and ACS. Furthermore, during infection, there is an increased metabolism related with fever and tachycardia, which in addition to vascular effects, can contribute to ACS development [10,11]. Additionally, previous studies found a relation between the occurrence of ACS and temperature changes, both daily and monthly [12]. The decrease in temperature and increase in atmospheric pressure were associated with an increased occurrence of ACS [13]. Thus, the study aimed to assess risk factors for coronary artery thrombosis as a manifestation of ACS (one of the myocardial infarction type 1 mechanisms), including air pollution, meteorological data, and LRTIs. The secondary aim included a search for potential risk factors of LRTI in patients with ACS. 

## 2. Materials and Methods

### 2.1. Study Design

**Figure 1:**
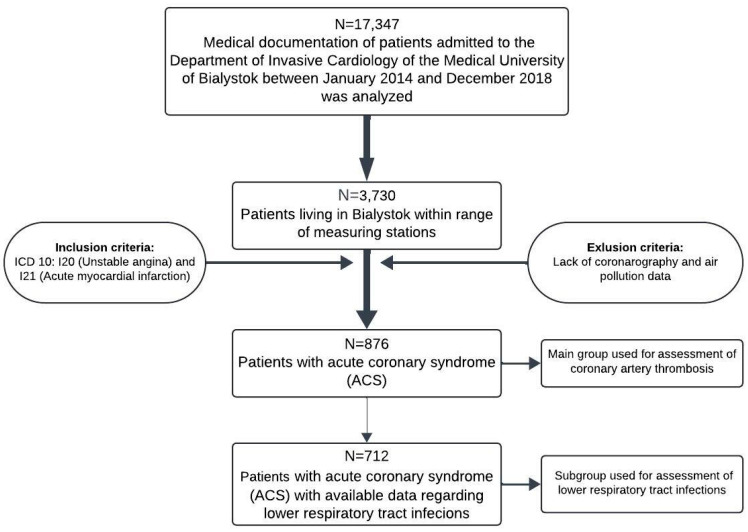
Study design flowchart.

We performed an analysis of the medical records of 876 patients with ACS (ICD-10: I20 or I21) in whom coronary thrombosis could be evaluated (as presented in Figure 1). The exclusion criteria were a lack of available data on coronarography and air pollution.

ACS was defined as a myocardial infarction (MI) in accordance with the universal definition of an unstable angina. MI was defined as a myocardial injury (rise and/or fall of troponin with at least one value over the 99th percentile) with evidence of acute myocardial ischemia (symptoms, new ischemic changes in ECG, development of Q waves, loss of myocardial motion, or identification of a coronary thrombus in an angiography) [14].

The calculated sample size with a 5% margin of error and confidence level of 95% was 385 (Sample Size Calculator: calculator.net accessed on 29 July 2024). The analysis included medical history, basic laboratory tests, clinical data, and coronary angiographies. Coronary artery thrombosis was defined as a thrombus present in a coronarography or in an intravascular ultrasound (IVUS) [15,16]. The angiographic signs of coronary thrombosis include Ambrose complex lesion morphology, a spherical, ovoid, or irregular filling defect, abrupt vessel cutoff, and intraluminal staining [17]. The LTRI was defined as an infection developed a day before or after admission and included mainly bronchitis (ICD10—J20) and pneumonia (ICD10—J18). LRTIs were clinically diagnosed by the physicians, based on the symptoms, inflammatory markers, and chest X-ray results. Data sufficient to assess for clinically noticeable LRTI were available for 712 patients and that population was used as a subgroup in the analysis of LRTI. The analyzed comorbidities were taken from medical documentation and were defined according to the current European guidelines. The laboratory tests were performed at the Medical University of Bialystok Clinical Hospital. The white blood cell (WBC) count was assessed by a direct current detection method with coincidence correction using a Sysmex Hematology Analyzer (Sysmex, Kobe, Japan). The concentration of C-reactive protein (CRP) in serum was determined using the turbidimetric method with Alinity I. The concentration of fibrinogen was analyzed using the Clauss method with an STA R Max 2 Coagulation Analyzer (Diagnostica Stago, Düsseldorf, Germany). The meteorological data were gathered from a local measuring center in Bialystok.

### 2.2. Air Pollution Data

The local air quality parameters, over 3 days before admission of each patient, were obtained from the Chief Inspectorate of the Environmental Protection of Poland. The measurements from the two measuring stations in Bialystok (Waszyngtona Street 16 station—code: PL0148A; Warszawska Street 75A station—code: PL0496A) were included. The analyzed parameters were 24-h daily average values of NO_2_, SO_2_, and CO, particulate matter with a diameter of 2.5 μm or less (PM_2.5_) and particulate matter with a diameter of 10 μm or less (PM_10_).

### 2.3. Statistical Analysis

Continuous variables were expressed as median ± standard deviation. Categorical variables were expressed as percentages (number of patients). The normal distribution was tested using the Kolmogorov–Smirnov test. Statistical tests used for analysis included Student’s *t*-test for parametric continuous variables, the Mann–Whitney U-test for non-parametric continuous categorical variables, and the Chi-squared test and odds ratio Altman calculation for categorical variables. The statistics were performed using Statistica 13. The logistic regression was performed using Stata 18.0. *p*-value ≤ 0.05 was considered as significant. The pairwise deletion approach was used in missing data curation. 

### 2.4. Ethics

The study was conducted in accordance with the Declaration of Helsinki and approved by the Institutional Review Board of the Medical University of Białystok (approval number APK.002.81.2022; the date of approval: 10 February 2022). Informed consent was obtained from all subjects involved in this study.

## 3. Results

### 3.1. General Population

The median age of subjects was 68 years and 63.24% of the subjects were male. The most common comorbidities were hyperlipidemia and hypertension (Table 1). In total, 9.13% (*n* = 65) patients with ACS suffered from LTRI and 13.01% had coronary artery thrombosis. In total, 32.5% of the patients had UA, 39.1% had NSTEMI, and 28.4% had STEMI.

### 3.2. Coronary Artery Thrombosis

There were no statistically significant differences in age, body mass index (BMI), CRP concentrations, and fibrinogen concentrations between the patients with and without coronary artery thrombosis. However, patients with coronary thrombosis had a higher WBC count (10.22 ± 4.62 vs. 8.43 ± 6.18; *p* < 0.0001) and a higher prevalence of LRTI (17.58% vs. 7.89%; odds ratio: 2.4903; 95% CI: 1.3483 to 4.5996; *p* = 0.0036). Furthermore, smoking tended to be more often connected with coronary thrombosis (35.56% vs. 26.04%; odds ratio: 1.5668; 95% CI: 0.9806 to 2.5033; *p* = 0.06). Both hypertension (53.85% vs. 76.61%; odds ratio: 0.3561; 95% CI: 0.2266 to 0.5597; *p* < 0.0001) and diabetes mellitus (17.58% vs. 31.61%; odds ratio: 0.4615; 95% CI: 0.2621 to 0.8125; *p* = 0.0074) occurred less frequently among subjects with coronary thrombosis than without coronary thrombosis (Table 2). The prevalence of coronary artery thrombosis was 0% for UA, 6% for NSTEMI, and 18.6% for STEMI (*p* < 0.0001). 

There were no statistically significant differences in NO_2_, SO_2_, CO, PM_2.5_, and PM_10_ concentrations over the 3-day period preceding ACS in patients with and without coronary thrombosis. However, there was a trend between a lower atmospheric pressure and patients with coronary thrombosis (Table 3). Additionally, in a subanalysis including the type of ACS, the atmospheric pressure was significantly lower before STEMI in patients with coronary thrombosis (994 vs. 999 hPa; *p* = 0.0078). Analysis using logistic regression did not find any significant effect on coronary artery thrombosis (Table 4). However, in patients with STEMI, the increase in atmospheric pressure was connected with a lower probability of coronary artery thrombosis (Table 5).

### 3.3. Lower Respiratory Tract Infections (LRTIs)

Patients with LTRI were older (80.00 ± 14.00 years vs. 67.00 ± 12.26 years; *p* < 0.0001), had lower BMI values (25.60 ± 4.65 years vs. 28.99 ± 4.97 years; *p* = 0.0102), and had higher inflammatory markers: WBC count (*p* < 0.0001), CRP concentration (*p* < 0.0001), and fibrinogen concentration (*p* = 0.0173). The prevalence of atrial fibrillation was higher among patients with LRTI than without LTRI (39.68% vs. 17.00%; odds ratio: 3.2117; 95% CI: 1.8626 to 5.5380; *p* < 0.0001). There was a statistically non-significant trend towards a higher rate of diabetes mellitus (39.68% vs. 28.75%; odds ratio: 1.6306; 95% CI: 0.9572 to 2.7777; *p* = 0.0720) and a lower rate of hyperlipidemia (58.73% vs. 68.93%; odds ratio: 0.6413; 95% CI: 0.3781 to 1.0880; *p* = 0.0995) and hypertension (65.08% vs. 74.50%; odds ratio: 0.6380; 95% CI: 0.3691 to 1.1028; *p* = 0.1075) in subjects with LRTI. Furthermore, COPD tended to occur more frequently in patients with LRTI than without LTRI (9.52% vs. 5.26%; odds ratio: 1.8978; 95% CI: 0.7644 to 4.7118; *p* = 0.1673) (Table 6). The prevalence of LRTI was 0.6% for UA, 8% for NSTEMI, and 17% for STEMI (*p* < 0.0001).

Patients with LRTI were exposed to a higher concentration of PM_2.5_ over three consecutive days before hospitalization due to ACS (16.35 µg/m^3^ vs. 13.15 µg/m^3^; *p* = 0.0402). Additionally, without statistical significance, PM_10_ tended to be higher over three consecutive days before hospitalization (24.63 ± 14.03 µg/m^3^ vs. 21.48 ± 13.67 µg/m^3^; *p* = 0.1508). There was also a trend towards a higher concentration of NO_2_ (13.72 ± 6.11 µg/m^3^ vs. 12.08 ± 5.60 µg/m^3^; *p* = 0.0967), PM_10_ (23.74 ± 17.30 µg/m^3^ vs. 21.48 ± 13.67 µg/m^3^; *p* = 0.2536), and CO (0.35 ± 0.13 mg/m^3^ vs. 0.33 ± 0.13 mg/m^3^; *p* = 0.1396) one day before hospitalization (Table 7). The multivariable logistic regression showed that the increase in PM_2.5_ in the past three days corresponded to a higher risk of LRTI (Table 8).

## 4. Discussion

### 4.1. Air Pollution and Infections

Our study shows the previously described relation between the increased rate of respiratory tract infections and the exposition to higher levels of PM_2.5_, focusing on patients with ACS. Previous studies indicated that elevated PM levels increase both the susceptibility and severity of infections with respiratory viruses through the strong induction of oxidative stress [18,19,20]. PM_2.5_ particles are able to reach to the lowest portions of the respiratory tracts and alveoli, accumulate there, and induce injury to the epithelial barrier of the bronchioles and alveoli, predisposing individuals to microbial invasion and promoting epithelial inflammation [8]. PM_2.5_ not only activates the innate type of immunity by promoting inflammation markers, but also predisposes to infections. The relationship between acute lower respiratory tract infections and the increase in PM_2.5_ concentration in a week preceding hospitalization was found in a group of 146,397 patients from an observational case-crossover designed study. However, the largest increase in admissions was noted after 3 weeks [21]. Furthermore, the past increases in CO, SO_2_, and PM_2.5_ concentrations were connected with upper respiratory tract infections and pneumonia occurrence [22]. The other study showed a significant association between environmental factors, such as temperature, humidity, and the concentration of NO_2_, O_3_, PM_10_, and CO, and an increased number of COPD exacerbations. However, the authors pointed out some limitations of the mentioned study; they have neither included influenza morbidity data nor PM_2.5_ pollution. We observed a non-significant tendency of a slightly increased number of LRTIs in the group with COPD [23].

In a study by Chen et al., 10.7% cases of influenza were suggested to be a result of exposure to ambient PM_2.5_. The strongest effect was observed at lag day two (RR: 1.015; 95% CI: 1.004, 1.025; per 10 μg/m^3^ increase in PM_2.5_) [24]. Similarly, in our study, the statistically higher concentration of PM_2.5_ was only noticed when the 3 days of exposure were included. Additionally, the impact of air pollution is not only limited to the short-exposure trigger. In a nationwide test-negative case-controlled study in the Netherlands, the short-term exposure to PM_10_ (adjusting for NO_2_) and PM_2.5_ was positively correlated with the increased odds of testing positive for SARS-CoV-2. A similar relation was observed in the long-term exposure to PM_10_ and PM_2.5_ [25]. A study by Kaspersen et al. showed that PM_2.5_ maintains significant influence on the incidence of respiratory tract infections within a wide range of time exposure (from 3 days to 12 months) [26]. 

### 4.2. Infection and Hypercoagulability

The higher rate of coronary artery thrombosis in the course of acute coronary syn-drome in patients with LRTI is the main finding of this investigation, which confirms previous observations. Acute infections cause transient endothelial dysfunction, increased pro-inflammatory markers, and shift the lipid profile towards pro-atherogenic [27]. It promotes plaque instability, platelet activation, and blood hypercoagulability. Community-acquired pneumonia is frequently complicated by myocardial infarction, that involves excessive platelet activation, as suggested by the increased thromboxane concentrations [28,29].

Therefore, respiratory infections increase the risk of myocardial infarction in the short term (OR = 17.0, 95% CI: 13.2–21.18) [30]. Similar conclusions are presented in the meta-analysis by H. Roi-Teeuw et al., which showed an increased cardiovascular risk, including ACS (OR = 2.9; 95% CI 1.8–4.9), in the presence of respiratory infections. The study included infections of various etiologies, both bacterial and viral, as well as chronic and acute inflammation of the upper and lower respiratory tract [31]. Additionally, a study by Kwong et al. showed that respiratory infections increase the risk of ACS in 7 days following the diagnosis (RR: 6.05; 95% CI: 3.86 to 9.50) with the highest impact on influenza B (RR: 10.11; 95% CI: 4.37 to 23.38) [32]. Furthermore, in our study, the patients with coronary thrombosis were less likely to be subjected to classical risk factors such as diabetes mellitus and hypertension. The previous studies connected hypercoagulation with poor glycemic control and extreme hyperglycemia [33]. We suspect that coronary thrombosis can be more likely correlated with dynamic changes, rather than with the diagnosis itself. Thus, infection as a non-classical risk factor can be crucial in the pathogenesis of coronary thrombosis. However, prospective studies are required in order to verify this hypothesis. 

The above-mentioned data allow a consideration of vaccines as a part of the prevention of cardiovascular complications. A randomized, double-blind, placebo-controlled, multicenter trial conducted by Fröbert et al. aimed to investigate the impact of influenza vaccination within 72 h after myocardial infarction or in high-risk CHD patients on all-cause and cardiovascular mortality. Of the 2532 patients, 1272 received influenza vaccination and 1260 received a placebo. The study showed a significant 41% reduction of all-cause and cardiovascular death risks and a trend towards the reduced risk of recurrent infarction among vaccinated patients [34].

The second trial, with influenza vaccination after myocardial infarction (IAMI), showed that vaccines improve the prognosis, especially in the group of patients with NSTEMI when compared to STEMI. The primary endpoint, consisting of all-cause death, MI, or stent thrombosis at 12 months post-randomization, in the NSTEMI group occurred in 6.5% vs. 10.5% of the group assigned to influenza vaccine vs. the group assigned to the placebo (HR = 0.60; 95% CI, 0.39–0.91), compared to 4.1% assigned to the influenza vaccine and 4.5% assigned to the placebo in the STEMI group (HR = 0.90; 95% CI, 0.54–1.50, *p* = 0.237) [35]. 

Additionally, recent ESC guidelines regarding ACS suggest that despite infections, systemic and local inflammation should be investigated as a treatment target in patients with atherosclerosis [1]. High-sensitivity CRP increases in patients with STEMI with a peak within 48–72 h [36]. An elevation in hs-CRP occurred 30 days after MI was associated with poorer prognosis [37]. The increased systemic immune–inflammation index (SII, platelet × neutrophil/lymphocyte ratio) was connected with a decrease in ejection fraction and an increase in the number of all-cause and cardiovascular deaths in patients with MI [38]. Furthermore, the systemic inflammation response index (SIRI) was also associated with left ventricular adverse remodeling after MI [39]. The increased myocardial tissue inflammation expressed as increased T2 values of the non-infarcted myocardium (NIM) in cardiac magnetic resonance, after primary percutaneous coronary intervention for STEMI, was related to a higher rate of myocardial reinfarction and a larger infarct size [40]. Canakinumab is a therapeutic monoclonal antibody targeting interleukin-1β. The specific treatment for patients with previous myocardial infarction and elevated high-sensitivity CRP with a 150-mg canakinumab dose caused a decrease in recurrent cardiovascular events [41]. However, such an approach led to an increase in serious infections and was not proven to improve all-cause mortality. The early, intensive, lipid-lowering therapy remains a key aspect of ACS treatment, as it causes not only ameliorate lipid concentration, but also achieves lipid-independent anti-inflammatory effects [42,43]. The initiation of the treatment with a high intensity statis of ACS in addition to ezetimibe and/or Proprotein Convertase Subtilisin/Kexin Type 9 Inhibitors should be considered [1,44].

### 4.3. Air Pollution and Cardiovascular Diseases

Air pollution is considered a significant risk factor of cardiovascular diseases by the European Society of Cardiology Guidelines on cardiovascular disease prevention in clinical practice released in 2021. The loss of life expectancy due to ambient air pollution has been estimated for 2.9 years. Considering that up to one-third of Europeans living in urban areas are exposed to levels exceeding the European Union’s air quality standards, it is one of the major public health issues [45]. The air pollution can cause ACS in patients without classical risk factors and the knowledge of life-time exposure to air pollution should be taken into consideration when assessing the cardiovascular risk. 

Other cardiovascular diseases potentially induced by air pollution are arrhythmias, especially atrial fibrillation (AF). Although the pathophysiological mechanism remains unclear, a study involving 190,115 patients with acute onset of symptomatic arrhythmia showed an increased risk in the onset of symptomatic arrhythmia within the first hours of exposure. However, after 24 h, this risk substantially attenuated, and an increase in PM_2.5_, NO_2_, SO_2_, and CO concentrations was associated with a significantly higher risk of atrial fibrillation (1.7–3.4%), atrial flutter (8.1–11.4%), and supraventricular tachycardia (3.4–8.9%) [46]. Such effects of PM_2.5_ were confirmed by other studies [47,48,49]. Air pollution may also worsen the prognosis and may increase the frequency of hospital admissions due to AF, ischemic stroke, and heart failure [50,51,52,53,54]. In our study, the patients with LRTI who were more exposed to PM_2.5_ had a higher rate of AF (39.68% vs. 17.00%). Considering the known relationship between infections and AF, more studies should investigate infections as a potential cause for air pollution-related episodes of AF. 

In the study by Kuźma et al., an 8% increase in admissions due to ACS was linked to an increase in NO_2_ concentration. However, no association was found between concentrations of PM_2.5_, PM_10_, and SO_2_ and the frequency of hospitalizations were related to heart disease. The authors speculate, that due to the relatively low level of air pollution during the limited period of the study, in the urban region of Bialystok, the burden of exposure to these pollutants might be insufficient to induce a higher mortality/hospitalization rate [55]. In another study conducted in Krakow, Poland, which is characterized by significantly high air pollutant concentrations, the 10-μg/m^3^ increase in PM_2.5_ was associated with a higher risk of myocardial infarction hospitalization (OR = 1.32, 95% CI: 1.01–1.40, *p* < 0.001) [56]. Also, exposure to PM_10_ was reported to influence the prevalence of ACS. In the report from the same center (Krakow, Poland), the impact of PM_10_ concentration on the number of ACS patients and the associated PCI in polluted and non-polluted regions was analyzed. An increase in PM_10_ concentration by 1 μg/m^3^ increased the rate of angioplasty in patients with ACS by 0.22 and 0.18 per week, respectively, in the group of patients from the non-polluted vs. polluted area [57]. The influence on SO_2_ and PMs was also confirmed in another study comparing the frequency of air pollution-related ACS in the industrial and non-industrial regions of Poland. It showed a relationship between the increase in the concentration of these particles and the increase in the frequency of ACS in the industrial area. It also confirmed the effect of NO_2_ in both regions, especially on the incidence of NSTEMI [32]. Noteworthy is also the multicenter longitudinal study ELAPSE, evaluating seven population-based cohorts. The study revealed a statistically significant, positive correlation between the exposure to 5 μg/m^3^ PM_2.5_ (OR = 1.041, 95% CI: 1.010–1.072), 10 μg/m^3^ NO_2_ (OR = 1.025, 95% CI: 1.006–1.044), and 0.5 × 10^−5^/m^3^ black carbon (OR = 1.022, 95% CI: 1.004–1.040) and cardiovascular mortality [58]. Furthermore, in the statistical analysis of 80 million patient years, the air pollution was related to the occurrence of NSTEMI and STEMI, especially in younger people, women, residents of rural areas, and those with a lower socioeconomic status. Additionally, reduction to the WHO norm could prevent a significant number of ACS cases [6]. The time correlation between the acute plaque change and air pollution was evaluated by Rinaldi et al. They assessed the mechanism of plaque instability using intracoronary optical coherence tomography and showed that among patients with ACS, on the day of the incident, patients with a ruptured atherosclerotic plaque coincided with a higher PM_2.5_ concentration. The analysis showed that this was the only statistically significant, independent factor influencing plaque rupture. The event was preceded by an increase in PM_2.5_ concentrations in the previous days, with the highest values on the day of ACS [59]. Furthermore, PM_2.5_ is associated with the increase in cardiovascular mortality [60,61]. Additionally, even in cases of non-obstructive coronary artery disease, air pollution was associated with a higher risk of epicardial spasm and myocardial infarction with non-obstructive coronary arteries [62]. In the present study, the prevalence of the classical atherosclerosis risk factors, such as hypertension and diabetes mellitus, was lower in patients with coronary artery thrombosis. There was a significantly higher prevalence of LRTI among patients with coronary artery thrombosis. As air pollution is related to LRTI, we also suspect that it may be involved in the pathogenesis of acute plaque rupture and coronary artery thrombosis via the promotion of infections and inflammation. 

### 4.4. Atmospheric Pressure and Cardiovascular Diseases

In our study, the atmospheric pressure was significantly lower a day before hospitalization in patients with coronary thrombosis during STEMI, with a similar tendency in the general population. In previous studies, the decrease in atmospheric pressure was associated with a higher risk of deep venous thrombosis [63]. Furthermore, Hong et al. found that acute changes in atmospheric pressure increased the incidence of STEMI (exposure over 7 days before hospitalization) [64]. However, the study by Didier et al. showed that an increase in atmospheric pressure was positively associated with the weekly higher incidence of STEMI [65].

### 4.5. The Influence of Malnutrition and Age on Infections

Our study showed that the patients with LRTI had lower BMI (25.60 ± 4.65 vs. 28.99 ± 4.97; *p* = 0.0102). Increased risk factors may be attributed to potential malnutrition, as reflected in previous studies [66,67]. However, this index imperfectly reflects nutritional status, suggesting that assessing the MUST index (Malnutrition Universal Screening Tool), which incorporates clinical evaluation and the percentage of weight loss alongside BMI, could be a more reliable direction for further research [67]. Furthermore, in our study, the patients with LRTI were older and also the previous studies connected malnutrition and a higher risk of infection with the elderly [68].

## 5. Study Limitations

### 5.1. Study Design

The study was performed at a single center, limited to patients from the single city with two air pollution measuring stations. It was performed in a region with low-to-medium air pollution; thus, the results cannot be generalized to high-pollution areas. The personal direct influence of air pollution was not analyzed. The data regarding air pollution were assessed for specific parts of the city. The study was a retrospective analysis, thus, the causality relation between variables may be limited. The retrospective nature of the analysis does not allow the calculation of the risk of ACS in LRTI patients, however the odds ratio that is included provides a reasonable approximation of the relative risk.

The subjects originated from Eastern Europe and all participants were Caucasian, so the results cannot be generalized to other ethnicities.

### 5.2. Data Collection

Due to the data collected from the medical documentation and air pollution monitoring measuring stations, the lack of data does not allow including the whole initial population, limiting the sample size. Furthermore, the monitoring stations were localized in the center of the city.

## 6. Future Directions

There is a need for further studies regarding possible mechanisms connecting air pollution and ACS. The relationship between air pollution infections and ACS should be investigated in prospective studies. The usefulness of preventive measures, such as face masks and pneumococcal, influenza, and RSV vaccines, in the regions of significant air pollution should be studied. 

## 7. Conclusions

The occurrence of coronary artery thrombosis was higher among subjects with LRTI during ACS. PM_2.5_ exposition was higher over three consecutive days before the hospitalization of patients with LRTI during ACS. 

## Figures and Tables

**Table 1:** General characteristics of study population.

Study Population *n* = 876			
Age	68.00 ± 12.64 years	COPD	4.68% (41)
Male	63.24% (554)	Diabetes mellitus	24.20% (212)
Smoking	21.46% (188)	Atrial fibrillation	15.64% (137)
Hypertension	59.82% (524)	Hyperlipidemia	55.14% (483)
NO_2_ [µg/m^3^]	12.32 ± 5.45	PM_2.5_/3 days [µg/m^3^]	13.88 ± 11.74
SO_2_ [µg/m^3^]	3.17 ± 2.25	PM_10_/3 days [µg/m^3^]	21.50 ± 13.47
CO [mg/m^3^]	0.33 ± 0.13	Temperature [°C]	7.9 ± 8.05
PM_2.5_ [µg/m^3^]	12.31 ± 13.60	Atmospheric pressure [hPa]	997.6 ± 8.18
PM_10_ [µg/m^3^]	20.19 ± 16.59		

**Table 2:** Comparison between patients with and without coronary artery thrombosis.

*n* = 876	With Coronary Thrombosis (114)	Without Coronary Thrombosis (762)	*p* Value
Age [years]	68.00 ± 13.32	68.00 ± 12.52	0.2312
Male	64.91% (74)	62.99% (480)	0.6917
Smoking	35.56% (32)	26.04% (156)	0.0604
COPD	4.40% (4)	5.97% (37)	0.5496
BMI	28.36 ± 5.67	28.76 ± 4.89	0.7139
LRTI	17.58% (16)	7.89% (49)	0.0036
WBC [1000/μL]	10.22 ± 4.62	8.43 ± 6.18	<0.0001
CRP [mg/L]	11.05 ± 69.77	6.30 ± 48.28	0.1373
Fibrinogen [mg/dL]	371.00 ± 122.35	373.00 ± 105.67	0.9124
Diabetes mellitus	17.58% (16)	31.61% (196)	0.0074
Hypertension	53.85% (49)	76.61% (475)	<0.0001
Atrial fibrillation	18.68% (17)	19.32% (120)	0.8846
Hyperlipidemia	68.13% (62)	68.01% (421)	0.9819

**Table 3:** The air pollution and meteorological data before hospitalization; comparison between patients with and without coronary artery thrombosis.

*n* = 876	With Coronary Thrombosis (114)	Without Coronary Thrombosis (762)	*p* Value
NO_2_ [µg/m^3^]	11.97 ± 5.48	12.48 ± 5.61	0.2637
SO_2_ [µg/m^3^]	3.25 ± 1.49	3.13 ± 2.41	0.6699
CO [mg/m^3^]	0.33 ± 0.13	0.33 ± 0.12	0.5903
PM_2.5_ [µg/m^3^]	13.35 ± 11.66	12.69 ± 13.29	0.8600
PM_10_ [µg/m^3^]	19.62 ± 14.39	20.80 ± 16.36	0.5867
PM_2.5_/3 days [µg/m^3^]	14.46 ± 9.31	13.90 ± 11.68	0.7804
PM_10_/3 days [µg/m^3^]	21.55 ± 10.95	21.41 ± 13.49	0.7817
Temperature [°C]	5.80 ± 7.67	8.05 ± 8.08	0.5497
Atmospheric pressure [hPa]	996.4 ± 9.27	998.0 ± 8.03	0.0641

**Table 4:** The multivariable logistic regression analyzing the impact on coronary artery thrombosis.

General Population Coronary Thrombosis	Odds Ratio	SE	*p* Value	[95% Conf. Interval]
NO_2_ [µg/m^3^]	0.99	0.07	0.852	[0.86–1.13]
SO_2_ [µg/m^3^]	0.84	0.17	0.407	[0.56–1.26]
CO [mg/m^3^]	1.01	0.02	0.743	[0.96–1.05]
PM_2.5_ [µg/m^3^]	1.02	0.08	0.811	[0.87–1.20]
PM_10_ [µg/m^3^]	1.04	0.09	0.635	[0.88–1.24]
PM_2.5_/3 days [µg/m^3^]	1.01	0.11	0.892	[0.82–1.26]
PM_10_/3 days [µg/m^3^]	0.93	0.11	0.560	[0.73–1.18]
Temperature [°C]	1.00	0.05	0.955	[0.92–1.10]
Atmospheric pressure [hPa]	0.95	0.04	0.264	[0.87–1.04]

**Table 5:** The multivariable logistic regression analyzing the impact on coronary artery thrombosis in patients with STEMI.

STEMI Coronary Thrombosis	Odds Ratio	SE	*p* Value	[95% Conf. Interval]
NO_2_ [µg/m^3^]	0.88	0.09	0.221	[0.71–1.08]
SO_2_ [µg/m^3^]	0.68	0.22	0.238	[0.35–1.29]
CO [mg/m^3^]	1.00	0.03	0.989	[0.94–1.07]
PM_2.5_ [µg/m^3^]	0.97	0.16	0.851	[0.71–1.33]
PM_10_ [µg/m^3^]	1.17	0.21	0.373	[0.83–1.66]
PM_2.5_/3 days [µg/m^3^]	1.00	0.20	0.992	[0.67–1.49]
PM_10_/3 days [µg/m^3^]	0.97	0.218	0.880	[0.63–1.48]
Temperature [°C]	0.89	0.07	0.121	[0.76–1.03]
Atmospheric pressure [hPa]	0.88	0.05	0.037	[0.77–0.99]

**Table 6:** Comparison between patients with and without LRTI.

*n* = 712	With LRTI (65)	Without LRTI (647)	*p* Value
Age [years]	80.00 ± 14.00	67.00 ± 12.26	<0.0001
Male	58.46% (38)	64.30% (416)	0.3518
Smoking	28.33% (17)	27.19% (171)	0.8488
COPD	9.52% (6)	5.26% (34)	0.1673
BMI	25.60 ± 4.65	28.99 ± 4.97	0.0102
Coronary thrombosis	24.62% (16)	11.59% (75)	0.0036
WBC [1000/μL]	12.04 ± 5.95	8.42 ± 5.93	<0.0001
CRP [mg/L]	43.40 ± 66.97	5.10 ± 43.60	<0.0001
Fibrinogen [mg/dL]	410.00 ± 116.85	372.00 ± 105.26	0.0173
Diabetes mellitus	39.68% (25)	28.75% (186)	0.0720
Hypertension	65.08% (41)	74.50% (482)	0.1075
Atrial fibrillation	39.68% (25)	17.00% (110)	<0.0001
Hyperlipidemia	58.73% (37)	68.93% (446)	0.0995

**Table 7:** The air pollution and meteorological data before hospitalization; comparison between patients with and without LRTI.

*n* = 712	With LRTI (65)	Without LRTI (647)	*p* Value
NO_2_ [µg/m^3^]	13.72 ± 6.11	12.08 ± 5.60	0.0967
SO_2_ [µg/m^3^]	3.19 ± 1.69	3.16 ± 2.34	0.7695
CO [mg/m^3^]	0.35 ± 0.13	0.33 ± 0.13	0.1396
PM_2.5_ [µg/m^3^]	13.52 ± 15.28	12.58 ± 13.26	0.3378
PM_10_ [µg/m^3^]	23.74 ± 17.30	20.63 ± 16.44	0.2536
PM_2.5_/3 days [µg/m^3^]	16.35 ± 12.05	13.15 ± 11.68	0.0402
PM_10_/3 days [µg/m^3^]	24.63 ± 14.03	21.48 ± 13.67	0.1508
Temperature [°C]	10.2 ± 7.80	7.5 ± 8.08	0.6283
Atmospheric pressure [hPa]	999.3 ± 6.83	997.6 ± 8.03	0.1210

**Table 8:** The multivariable logistic regression analyzing the impact on LRTI.

General Population LRTI	Odds Ratio	SE	*p* Value	[95% Conf. Interval]
NO_2_ [µg/m^3^]	0.96	0.04	0.354	[0.88–1.05]
SO_2_ [µg/m^3^]	0.85	0.13	0.309	[0.62–1.16]
CO [mg/m^3^]	0.93	0.08	0.403	[0.79–1.10]
PM_2.5_ [µg/m^3^]	0.94	0.07	0.355	[0.81–1.08]
PM_10_ [µg/m^3^]	1.04	0.07	0.586	[0.91–1.19]
PM_2.5_/3 days [µg/m^3^]	1.04	0.019	0.035	[1.00–1.08]
PM_10_/3 days [µg/m^3^]	1.10	0.09	0.264	[0.93–1.29]
Temperature [°C]	0.97	0.03	0.328	[0.91–1.03]
Atmospheric pressure [hPa]	1.02	0.03	0.446	[0.96–1.09]

## Data Availability

The data presented in this study are available on request from the corresponding author.
